# The Changing Epidemiology of Coccidioidomycosis in Los Angeles (LA) County, California, 1973–2011

**DOI:** 10.1371/journal.pone.0136753

**Published:** 2015-08-27

**Authors:** Ramon E. Guevara, Tasneem Motala, Dawn Terashita

**Affiliations:** 1 Emergency Preparedness and Response Program, Department of Public Health, County of Los Angeles, Los Angeles, California, United States of America; 2 Acute Communicable Disease Control Program, Department of Public Health, County of Los Angeles, Los Angeles, California, United States of America; California Department of Public Health, UNITED STATES

## Abstract

Coccidioidomycosis, also known as Valley Fever, is often thought of as an endemic disease of central California exclusive of Los Angeles County. The fungus that causes Valley Fever, *Coccidioides* spp., grows in previously undisturbed soil of semi-arid and arid environments of certain areas of the Americas. LA County has a few large areas with such environments, particularly the Antelope Valley which has been having substantial land development. Coccidioidomycosis that is both clinically- and laboratory-confirmed is a mandated reportable disease in LA County. Population surveillance data for 1973–2011 reveals an annual rate increase from 0.87 to 3.2 cases per 100,000 population (n = 61 to 306 annual cases). In 2004, case frequency started substantially increasing with notable epidemiologic changes such as a rising 2.1 to 5.7 male-to-female case ratio stabilizing to 1.4–2.2. Additionally, new building construction in Antelope Valley greatly rose in 2003 and displayed a strong correlation (R = 0.92, Pearson p<0.0001) with overall LA County incidence rates for 1996–2007. Of the 24 LA County health districts, 19 had a 100%-1500% increase in cases when comparing 2000–2003 to 2008–2011. Case residents of endemic areas had stronger odds of local exposures, but cases from areas not known to be endemic had greater mortality (14% versus 9%) with notably more deaths during 2008–2011. Compared to the 57 other California counties during 2001–2011, LA County had the third highest average annual number of cases and Antelope Valley had a higher incidence rate than all but six counties. With the large number of reported coccidioidomycosis cases, multi-agency and community partnering is recommended to develop effective education and prevention strategies to protect residents and travelers.

## Introduction

Coccidioidomycosis, also commonly known as Valley Fever, is a fungal disease normally caused by the inhalation of airborne spores of *Coccidioides* spp. which grow in the soil of certain areas of the Americas, particularly in routinely hot, arid to semi-arid environments [1,2]. While an estimated 60% of infected people develop mild to no symptoms [3], the remaining infected present with various symptoms and conditions that can include weeks to months of fatigue, shortness of breath, cough, fever, night sweats, loss of appetite or weight, chest pain, headache, body aches, skin rash, and pneumonia [4]. Less than five percent of infected people develop disseminated disease which is when the fungus spreads beyond the lungs to infect any other body site such as skin, lymph nodes, bones, joints, and brain [4]. Disseminated disease can lead to life-long complications and death. An estimated 150,000 new infections occur each year in the United States [4], but reported cases from 2009 to 2013 ranged from 9,438 to 22,641[5]. In Arizona, 75% of reported cases miss work or school due to illness, and 40% of cases require hospitalization [6]. In California, the median cost of hospitalization alone is estimated to be U.S. $55,062 per patient [7]. As symptoms are nonspecific and disease awareness is low among primary healthcare providers [6,8], disease detection and timely treatment are major challenges.

At one time coccidioidomycosis was generally thought be acquired only in Central California of the United States and was commonly called San Joaquin Valley Fever [9]. Endemic areas have been recognized in parts of Central America, South America, and throughout the United States’ Southwest including Arizona, California, New Mexico, Nevada, Texas, and Utah [2,6,7,10]; however, awareness of coccidioidomycosis remains low, even in endemic areas [6,8]. For example, pockets of endemicity in San Fernando Valley of LA County have been documented since the 1950s [11–13], yet LA County remains an under-recognized source of Valley Fever.

**Fig 1:**
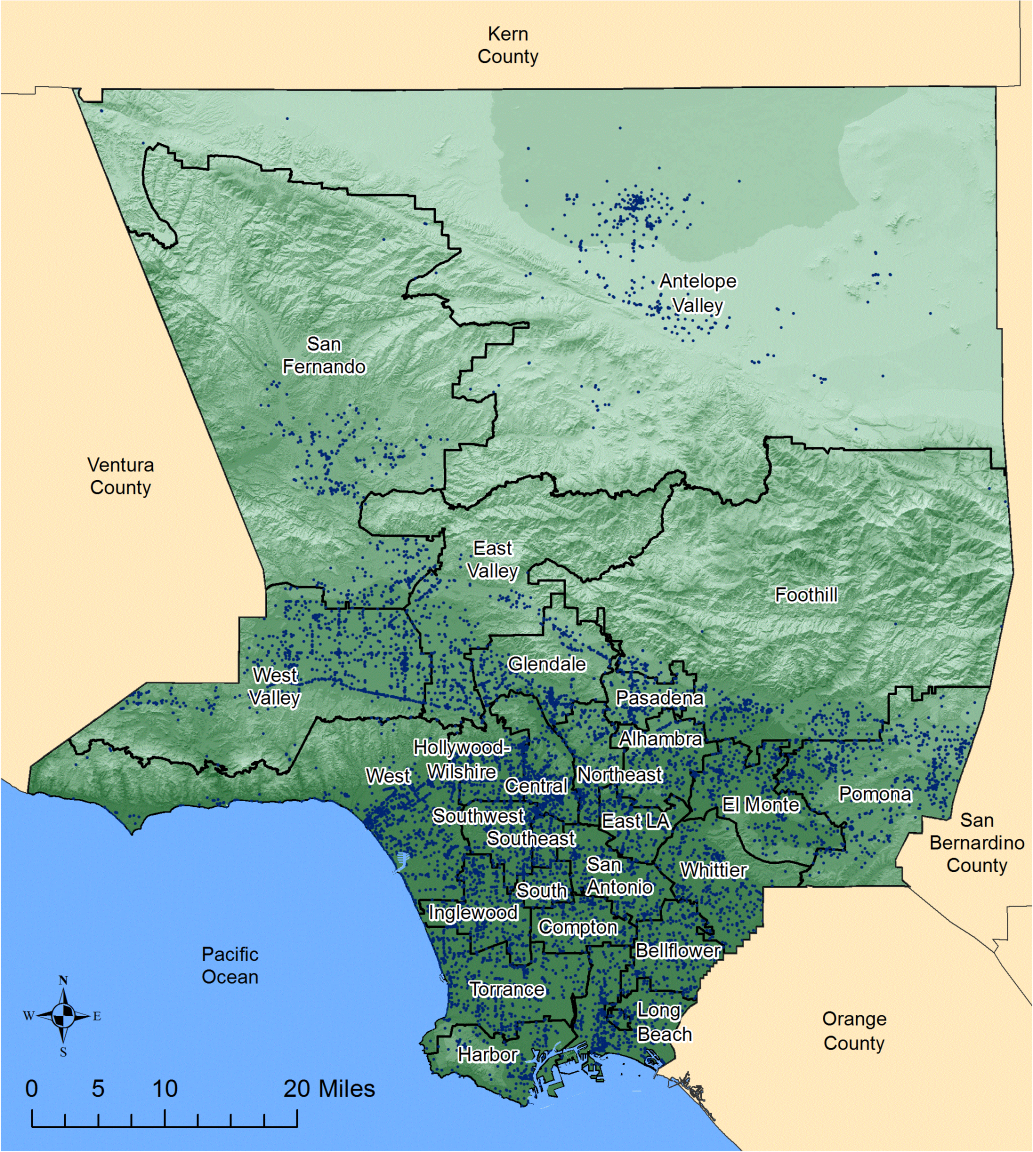
Geography of Los Angeles County, 2014. Legend. Land elevation is indicated by the shading of green with the lightest shade representing the highest elevation. The 24 health districts of LA County are indicated within their boundaries. Long Beach and Pasadena are cities outside LA County’s jurisdiction and have their own health departments. The dots represent urbanization as these are public facilities such as schools, libraries, post offices, bus stations, fire departments, police stations, museums, and hospitals. West Valley, San Fernando, and Antelope Valley districts are considered endemic for coccidioidomycosis based on environmental conditions and history of high case numbers and incidence rates. The high desert area of Antelope Valley continues to have the greatest potential for land development for projects such as housing, agriculture, and solar farms. Map was made by E.R. using ArcGIS 10.1 software.

LA County’s diverse geography of 4,084 square miles includes 70 miles of coastline, several deep valleys, mountains peaking over 10,000 feet, and a high desert area (Fig 1). With 10 million residents, a quarter of California’s population, LA County is the most populous county in the United States and more populous than most of the 50 U.S. states [14]. Most residents live in the lower elevation areas south of the mountains where urbanization is heaviest. Among the 24 county health districts, West Valley, San Fernando, and Antelope Valley are distinguished by large mountain valleys typically with higher summer temperatures, stronger winds, many more coccidioidomycosis cases, and higher incidence rates than the rest of the county. These three health districts are considered endemic for coccidioidomycosis while the other 21 health districts are not known to be endemic. Antelope Valley, a high desert area of 1,600 square miles at 2,270–3,500 feet above sea level [15] with very strong winds and dust storms, has the greatest potential in the county for land development projects such as housing, agriculture, and solar farms. Strong winds, dust storms, construction work, agriculture, solar farms, archaeological digs, and other soil disturbing activities have been previously associated with coccidioidomycosis cases and outbreaks [10,16–26]. Antelope Valley is directly south of Kern County, which traditionally has the greatest number of cases in California, and West Valley is southeast of Ventura County which experienced a coccidioidomycosis outbreak after a 1994 earthquake [27].

This study examines population-based surveillance data of 1973–2011 to present the epidemiology of coccidioidomycosis in LA County, California. Epidemiologic changes are analyzed together with urban development. Finally, LA County case numbers and rates are related to those of other California counties.

## Materials and Methods

The County of Los Angeles Department of Public Health has used the surveillance case definition of coccidioidomycosis of the Council of State and Territorial Epidemiologists (CSTE) [5,28–30] and has required health care providers and laboratories to report any identification of the disease since July 1, 1955. In 2008, the CSTE case definition was modified so that a single IgG positive test was sufficient for laboratory confirmation. Lacking clinical or laboratory evidence of disease, already existing in the surveillance database as a case, and being a non-resident of Los Angeles County were criteria for exclusion as new cases. Long Beach and Pasadena residents were excluded as these cities have their own health departments. Other noteworthy developments were the ability of local laboratories to report disease electronically starting in February 2002 and the addition of coccidioidomycosis to the state of California’s list of laboratory reportable diseases in December 2009 [31].

Case data from paper documents of annual disease summaries, which were available for 1973 and later, were compiled with electronic data since 1992 and analyzed with Microsoft Excel, Microsoft Access, and Statistical Analysis System software. Pearson correlation coefficients and Mantel-Haenszel formulas were used for statistical tests. Disease onset dates were established through medical records or case interview. Mutually exclusive race-ethnicity categories of Asian, black, Hispanic, and white were defined. Any identification of being Hispanic trumped all other race-ethnicity identifications. Designation of each case’s health district was routinely conducted to process investigation and follow-up of cases. Although data on travel history, occupation, and outdoor activities involving dirt were already being collected as part of routine surveillance, starting in 2005 additional exposure questions were added to case interviews to capture information on construction, earth excavation, dust storm, and other outdoor exposures within four weeks of symptom onset. Case interviews were conducted until February 2009 but data collection of exposures continued when such information was indicated in medical chart notes. Coccidioidomycosis-related mortality was defined by indication of death in the case report.

Other data sources included the United States Census Bureau for incidence rate calculations using 1990, 2000, and 2010 decennial counts, and for the number of building permits for new residential buildings constructed during 1996–2011 to measure urban development; Los Angeles County Vital Statistics population estimates for incidence rate calculations for years between the decennial census years; Los Angeles County Location Management System 2014 for geographic informational mapping; and the California Department of Public Health for case frequency and incidence rate comparisons among all the California counties.

The Institutional Review Board of the County of Los Angeles Department of Public Health (IRB) gave verbal consent of this study as the work performed is part of routine Department functions. The IRB waived written informed consent from participants because after de-duplication patient information was anonymized and de-identified prior to analysis.

## Results

### Overall trends

**Fig 2:**
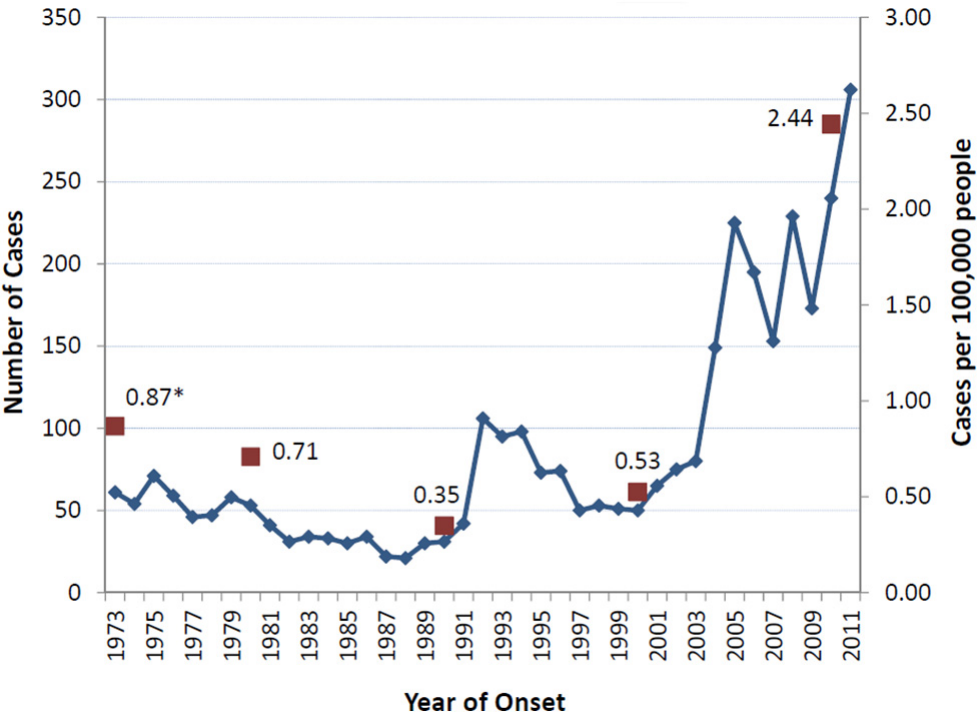
Annual coccidioidomycosis incidence (N = 3338) and decennial incidence rates, Los Angeles County, California, 1973–2011. Legend. Starting in 2004, substantial increases in the number of reported coccidioidomycosis cases occurred. Except for an outbreak during 1992 to 1994 involving strong winter storms and a 6.7 magnitude earthquake, annual cases numbered between 21 and 80 from 1973 to 2003. Sharp multi-year increases in cases occurred from 2003 (n = 80) to 2004 (n = 149) to 2005 (n = 225), and from 2009 (n = 173) to 2010 (n = 240) to 2011 (n = 306). Incidence rates were calculated using data from the U.S. Decennial Census. *A 1973 rate using the 1970 census count is presented because of the absence of surveillance data before 1973. These rates ranged between 0.35 and 0.87 cases per 100,000 people between 1973 and 2000, and increased 365% from 0.52 cases per 100,000 people in 2000 to 2.44 cases per 100,000 people in 2010.

Los Angeles County confirmed 3,338 reported coccidioidomycosis cases between 1973 and 2011. From 1973 to 2003, the number of reported coccidioidomycosis cases typically numbered between 21 and 80 per year (Fig 2). An outbreak during 1992 to 1994 that involved strong winter wind storms, the 1994 Northridge earthquake, and a larger outbreak seen in other California counties [27,32,33] briefly brought annual cases between 95 and 106. But a change started when annual case frequencies increased substantially from 80 in 2003 to 149 in 2004 and eventually to 306 in 2011. Incidence rate jumped 365% from 0.52 cases per 100,000 people in 2000 to 2.44 in 2010.

### Demographic Trends

#### Age

**Fig 3:**
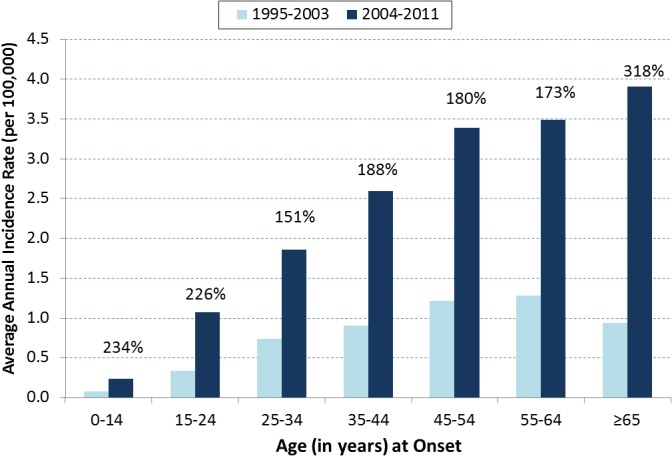
Average annual coccidioidomycosis incidence rate* by age (N = 2234), Los Angeles County, California, 1995–2011. Legend. *Average annual incidence rates were calculated by dividing the average annual number of cases for 1995–2003 and 2004–2011 by the U.S. Census population counts for 2000 and 2010, respectively. The period of 1992–1994 was excluded because these were outbreak years. For all age groups, average annual incidence rates increased substantially between 1995–2003 and 2004–2011. The percent increase ranged from 151% to 318%, with age groups ≥65, 0–14, and 15–24 years having the greatest increases. During 1995–2003, incidence rates increased with age until age group 55–64 years. However, during 2004–2011, incidence rates continued to increase in the age group ≥65 years.

Demographic aspects of the change starting in 2004 included the following. All age groups showed increasing cases after 2003, with many groups reflecting the steep spikes of the overall incidence in 2004, 2005, 2008, 2010, and 2011. During 1995–2003, average annual incidence rate increased with age until age 55–64 years (Fig 3). However, during 2004–2011, average annual incidence rate continued to increase with age at age ≥65 years. Between 1995–2003 and 2004–2011, age-specific average annual incidence rates increased 151%-318%. The greatest increases in rates occurred in the ≥65, 0–14, and 15–24 year age groups (318%, 234%, and 226%, respectively).

#### Race-Ethnicity

**Fig 4:**
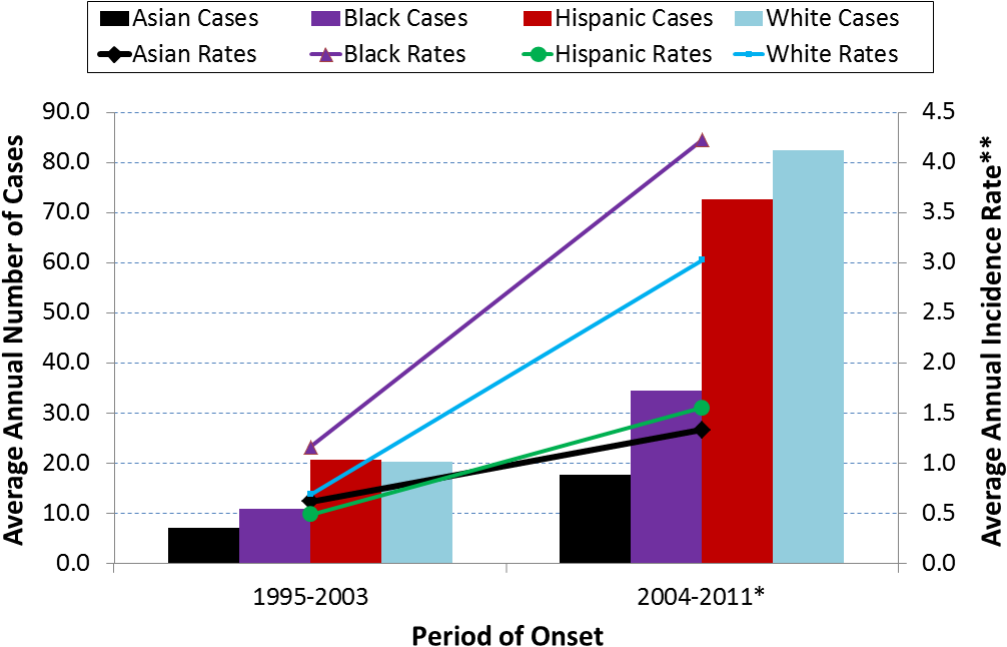
Average annual coccidioidomycosis incidence and incidence rates (IR) by race-ethnicity (N = 2234), Los Angeles County, California, 1995–2011. Legend. *2004–2011 excludes 2006–2008 because of substantial missing race-ethnicity data during those years and the missing data coincides with substantially less white cases. **Average annual incidence rates per 100,000 people were calculated by dividing the average annual number of cases for 1995–2003 and 2004–2011 (excluding 2006–2008) by the U.S. Census population counts for 2000 and 2010, respectively. Race-ethnicity categories are mutually exclusive. Between 1995–2003 and 2004–2011, all race-ethnicity categories had large increases in average annual incidence and in average annual incidence rate. The average annual number of cases increased from 7.0 to 17.6 for Asians (151%), 10.8 to 34.4 for blacks (219%), 20.6 to 72.6 for Hispanics (252%), and 20.3 to 82.4 for whites (306%). The average annual incidence rate per 100,000 people increased from 1.16 to 4.22 (264%) for blacks, 0.69 to 3.02 (338%) for whites, 0.48 to 1.55 (223%) for Hispanics, and 0.62 to 1.33 (115%) for Asians. Whites and Hispanics had the most number of annual cases on average, but blacks and whites had the highest incidence rates.

Regarding race-ethnicity, white and Hispanic cases rose sharply in 2004, 2005, and 2011 while increases among Asian and black cases were not as prominent. Usually 0%-15% of annual cases had missing race-ethnicity data. During 2006–2008, 36%-48% of cases were missing race-ethnicity data and the number of white cases substantially dropped. Between 1995–2003 and 2004–2011 (excluding 2006–2008 because of missing data), the average annual number of cases increased from 7.0 to 17.6 for Asians (151%), 10.8 to 34.4 for blacks (219%), 20.6 to 72.6 for Hispanics (252%), and 20.3 to 82.4 for whites (306%) (Fig 4). During 1995–2003, blacks, Hispanics, and whites had 1.54, 2.94, and 2.90 times higher numbers, respectively, compared to Asians. During 2004–2011, blacks, Hispanics, and whites had 1.95, 4.13, and 4.68 times higher numbers, respectively, compared to Asians. While whites and Hispanics led with much higher annual numbers on average, blacks led in average annual incidence rates. Between 1995–2003 and 2004–2011, average annual incidence rate (per 100,000 people) increased from 1.16 to 4.22 (264%) for blacks, 0.69 to 3.02 (338%) for whites, 0.48 to 1.55 (223%) for Hispanics, and 0.62 to 1.33 (115%) for Asians.

#### Gender

**Fig 5:**
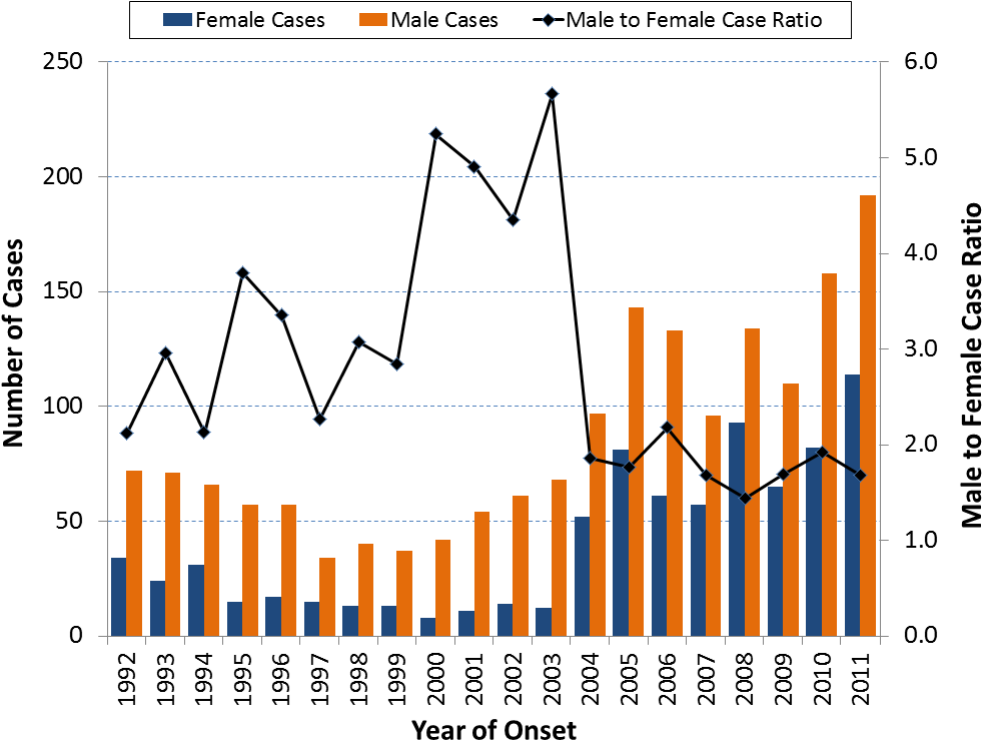
Coccidioidomycosis cases (N = 2534) by gender and male to female ratio, Los Angeles County, California, 1992–2011. Legend. The male to female ratio increased from 2.1 to 5.7 between 1992 and 2003. This changed starting in 2004 through 2011 as the ratio abruptly dropped and stabilized between 1.4 and 2.2. The number of female cases increased suddenly in 2004 but the number of male cases increased gradually since 1997.

The ratio of male to female cases rose from 2.1 to 5.7 between 1992 and 2003, but dropped and ranged from 1.4 to 2.2 between 2004 and 2011 (Fig 5). After the 1992–1994 outbreak, female cases numbered between eight and 17 per year until 2004 when it increased to 52. While the number of female cases increased suddenly in 2004 (333% from 12 in 2003), the increase in male cases was more gradual and started between 2000 and 2001. Male cases (n = 97) still had a substantial 43% increase in 2004 (n = 68 in 2003). Males and females in age groups 25–34 and 35–44 years experienced peak case numbers in 2005, but older age groups had trends of increasing cases over time (S2–S4 Figs). In all age groups, females had fewer cases than males (S1 Table). Within age groups, incidence trends of females generally reflected those of males (S1–S4 Figs).

### Geographic Trends

**Fig 6:**
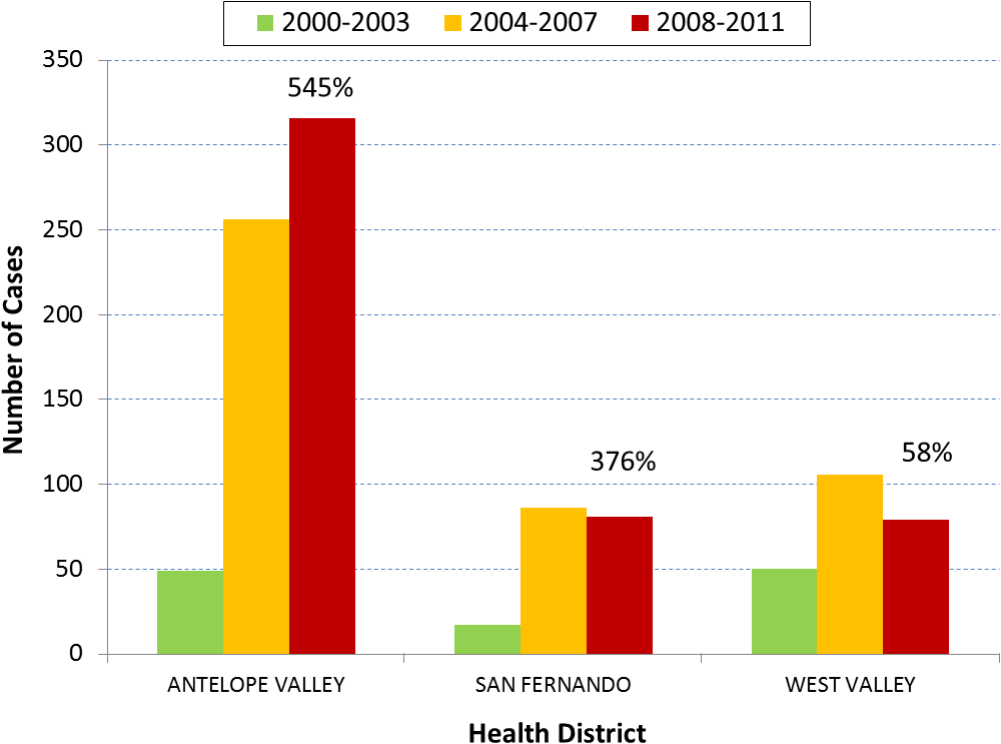
Coccidioidomycosis 4-year incidence (N = 1040) comparisons by endemic health districts of Los Angeles County, California, 2000–2011. Legend. Among the 24 health districts of Los Angeles County, Antelope Valley, San Fernando, and West Valley health districts are endemic for coccidioidomycosis as they have much higher case numbers traditionally as well as environments more favorable to *Coccidioides* spp growth. Year of disease onset was categorized into three 4-year time periods, 2000–2003, 2004–2007, and 2008–2011. For all three health districts, incidence sharply rose during 2004–2007. Unlike the other two health districts, Antelope Valley continued to see even more cases during 2008–2011. Percent increase in cases between 2000–2003 and 2008–2011 were 545%, 376%, and 58% for Antelope Valley, San Fernando, and West Valley, respectively.

**Fig 7:**
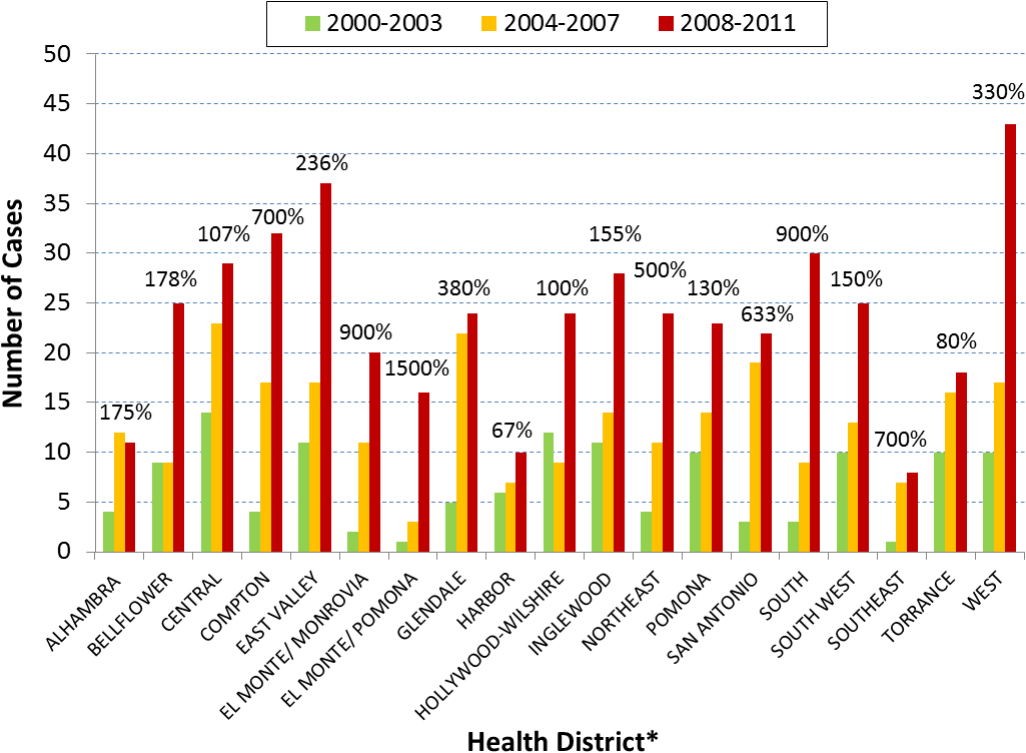
Coccidioidomycosis 4-year incidence (N = 903) comparisons by health districts not known to be endemic in Los Angeles County, California, 2000–2011. Legend. The increase in coccidioidomycosis cases has been extremely high in the health districts not known to be endemic in Los Angeles County. Year of disease onset was categorized into three 4-year time periods, 2000–2003, 2004–2007, and 2008–2011. *Only 19 of 21 health districts not known to be endemic are shown in the figure because East Los Angeles and Whittier had 14% and 18% decreases, respectively, between the first and last time periods. (For each successive time period, East LA had 7, 9, and 6 cases and Whittier had 11, 12, and 9 cases.) Case numbers rose steeply during 2004–2007 for at least eight health districts. For 18 health districts, case numbers rose steeply or continued to climb during 2008–2011. Percent increase in cases between 2000–2003 and 2008–2011 are shown and ranged between 67% and 1500%. Seventeen of the 21 health districts not known to be endemic had at least a 100% increase in the number of cases between 2000–2003 and 2008–2011.

Geographically, of the 24 health districts of Los Angeles County, 22 experienced an increase in cases during 2004–2007 and 19 during 2008–2011. Each of the three endemic health districts had many more cases than any of the individual health districts not known to be endemic (Figs 6 and 7). Of the 2,543 cases during 1992–2011, 1,215 (48%) resided in endemic health districts. During 1995–2003, endemic and not-known-to-be endemic health districts had 200 (40%) and 304 (60%) cases, respectively. During 2004–2011, there was a reversal of percentages as endemic and not-known-to-be endemic health districts had 924 (56%) and 735 (44%) cases, respectively. Between the two time periods, endemic health districts had a larger percent increase in cases (362%) than health districts not known to be endemic (142%). Antelope Valley had the greatest number of cases after 2000–2003 and unlike the other two endemic health districts it had more cases during 2008–2011 than 2004–2007 (Fig 6). Incidence rate (per 100,000 people) for Antelope Valley ranged from 2.27 to 5.79 during 2000–2003, 14.20 to 24.50 during 2004–2007, and 12.20 to 24.90 during 2008–2011. When comparing 2008–2011 to 2000–2003, Antelope Valley, San Fernando, and West Valley had 545%, 376%, and 58% more cases, respectively. Only two health districts not known to be endemic, East Los Angeles and Whittier, saw a decrease in cases between 2000–2003 and 2008–2011. All other health districts not known to be endemic experienced a percent increase in cases that ranged between 67% and 1500% (Fig 7). Seventeen of the 21 health districts not known to be endemic had case increases of 100% or more between 2000–2003 and 2008–2011.

### Mortality and Hospitalization

Mortality data ranged from 0% to 4% missing annually for 1997–2005 but 31% to 96% for other years during 1992–2011. Based on 1997–2005 data, coccidioidomycosis mortality was 11%, with 79 deaths among 724 cases with known survival status. During this period, mortality was 9% (33 deaths/385 cases) in endemic health districts and 14% (46 deaths/339 cases) in health districts not known to be endemic. During 2000–2003, 2004–2007, and 2008–2011, cases that died numbered 13, 18, and 17 in endemic health districts, and 21, 26, and 44 in health districts not known to be endemic.

Of 2,021 cases with hospitalization status data, 1,408 (70%) were hospitalized. During 1998–2009, when few cases were missing hospitalization status data (one to seven per year), the annual average of percent hospitalized was 68% with a range of 63%-79%.

### Exposures 1–4 Weeks Prior

Among measured exposures in the one to four weeks before illness, being in an area in sight of construction and being in an area in sight of earth excavation had the strongest associations with cases residing in endemic health districts (Table 1). Case residents of endemic areas had 5.48 (95% confidence interval: 3.87–7.75) times greater odds of being in an area in sight of construction and 5.46 (95% CI: 3.67–8.13) times greater odds of being in an area in sight of earth excavation within four weeks of illness onset than cases that resided in health districts not known to be endemic. After these two exposures, in order of diminishing magnitude, being in a dust storm, participating in outdoor activities involving recreational vehicles such as motorcycles and dirt bikes, participating in any outdoor recreation, participating in outdoor activities involving work with dirt, and having a job in an endemic health district had statistically stronger associations with cases that resided in endemic health districts. Conversely, travelling to an endemic area outside of Los Angeles County within four weeks before illness was more closely associated with cases that resided in health districts not known to be endemic. When focusing on cases without travel to endemic areas outside of LA County within four weeks of illness (n = 853), exposures regarding construction, earth excavation, dust storms, outdoor activity with dirt, and jobs in endemic areas in LA County became more strongly associated with case residents of endemic areas, and exposure by outdoor activities involving recreational vehicles became more associated with residents of health districts not known to be endemic.

**Table 1:** Exposures 1–4 weeks before coccidioidomycosis (N = 2543) by endemic/not-known-to-be endemic health district residency, Los Angeles County, California, 1992–2011.

Exposure	Residents With A “Yes” Response Over All Responders (%)			
	Endemic Health Districts	Not-Known-To-Be Endemic Health Districts	Total in LA County	Odds Ratio (95% Confidence Interval)
In sight of construction	247/377 (65.5%)	69/268 (25.7%)	316/645 (49.0%)	5.48 (3.87–7.75)
In sight of earth excavation	182/382 (47.6%)	38/266 (14.3%)	220/648 (34.0%)	5.46 (3.67–8.13)
Dust storm	142/352 (40.3%)	42/248 (16.9%)	184/600 (30.7%)	3.32 (2.24–4.92)
Outdoor recreational vehicles	21/380 (5.5%)	5/272 (1.8%)	26/652 (4.0%)	3.12 (1.16–8.39)
Any outdoor recreation	133/380 (35.0%)	64/272 (23.5%)	197/652 (30.2%)	1.75 (1.23–2.48)
Outdoor activity with dirt	155/789 (19.6%)	88/715 (12.3%)	243/1504 (16.2%)	1.74 (1.31–2.31)
Job in endemic area	66/1102 (6.0%)	39/1098 (3.6%)	105/2200 (4.8%)	1.73 (1.15–2.59)
Travel to endemic area outside of LA County	151/640 (23.6%)	223/587 (38.0%)	374/1227 (30.5%)	0.50 (0.39–0.65)

Except for travel to an endemic area outside of Los Angeles County, all the exposures listed above had statistically stronger associations with cases residing in endemic health districts. Travelling to an endemic area outside of Los Angeles County within four weeks before illness had a two-times statistically stronger association with cases residing in health districts not known to be endemic. Endemic health districts were Antelope Valley, San Fernando, and West Valley. Outdoor recreation vehicles included motorcycles, dirt bikes, and all-terrain vehicles. Outdoor activity with dirt specifically asked about yard work, landscaping, earth digging, building, outdoor house repair, and farming. When looking only at cases without travel to endemic areas outside of LA County (n = 853), effects of exposures regarding construction (OR = 7.87, 95% CI: 4.72–13.12), earth excavation (OR = 8.26, 95% CI: 4.41–15.48), dust storms (OR = 5.84, 95% CI 3.09–11.05), outdoor activity with dirt (OR = 2.05, 95% CI: 1.33–3.14), and jobs in endemic areas in LA County (OR = 2.72, 95% CI: 1.23–6.04) increased; however, the effect of any outdoor recreation decreased (OR = 1.48, 95% CI: 0.89–2.46) and the exposure of outdoor activities involving recreational vehicles became more associated with residents of health districts not known to be endemic (OR = 0.83, 95% CI: 0.23–3.01).

## Construction

**Fig 8:**
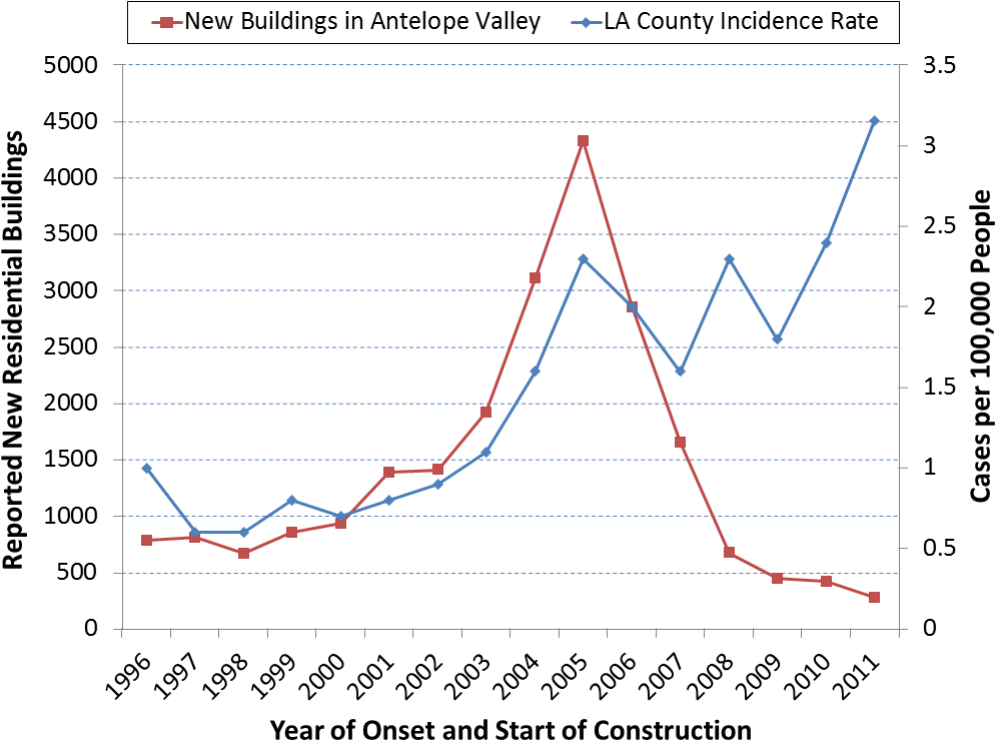
New residential buildings in Antelope Valley and coccidioidomycosis incidence rate in Los Angeles County, California, 1996–2011. Legend. The number of reported new residential buildings constructed in Antelope Valley was strongly correlated (R = 0.92, Pearson p<0.0001) with coccidioidomycosis incidence rate (per 100,000, N = 2168) in Los Angeles County during 1996–2007. The incidence rates here are based on population estimates from the 2000 U.S. census. Antelope Valley has had and continues to have the greatest potential for land development. The housing development boom of the early 2000s led to many residential neighborhood construction projects. Of all reported new residential buildings in Los Angeles County, 37%-39% during 2004–2005 versus 14%-23% during 1996–2003 were in Antelope Valley. Possible causal factors in higher incidence rates in 2008, 2010, and 2011 might include expansion of agricultural land, large land development for solar panels and wind farms, strong wind, the 2008 CSTE case definition modification of a single IgG positive test being confirmatory with signs and symptoms, and the 2009 addition of coccidioidomycosis to California’s list of laboratory reportable diseases.

Construction represented as the number of reported new residential buildings in Antelope Valley had a very strong correlation (R = 0.92, Pearson p<0.0001) with coccidioidomycosis incidence rate in LA County during 1996–2007 (Fig 8). Construction in other areas of the county did not have such a strong correlation with coccidioidomycosis. New residential buildings in Antelope Valley sharply increased from 1,929 in 2003 to 3,114 in 2004 and then peaked at 4,339 in 2005. Even earlier, construction greatly increased from 941 to 1,390 new residential buildings (48% increase) between 2000 and 2001, and from 1,415 to 1,929 (36% increase) between 2002 and 2003. Of all reported new residential buildings in Los Angeles County, 14%-23% during 1996–2003 versus 37%-39% during 2004–2005 were constructed in Antelope Valley. Although construction of new residential buildings decreased in Antelope Valley, the LA County annual coccidioidomycosis incidence rate never returned to pre-2004 levels. Rate increases in 2008, 2010, and 2011 indicated other factors were maintaining and increasing incident disease.

### Relative to Other California Counties

Compared to the 57 other counties in California during 2001–2011, LA County had the third highest average annual number of cases and Antelope Valley had a higher incidence rate than all but six counties (Table 2). Even during the more recent years of 2008–2011, LA County ranked high in number of cases and Antelope Valley ranked high in average annual incidence rate. While Kern, Fresno, and Kings Counties of the Central California Valley had the highest averages of annual cases and incidence rates during 2008–2011, other counties outside the Central California Valley such as San Luis Obispo, San Diego, Riverside, Orange, Ventura, San Bernardino, and Monterey had notably high averages of annual cases and incidence rates.

**Table 2:** Top average annual coccidioidomycosis cases and incidence rates (per 100,000 people) in California, 2001–2011 and 2008–2011.

	2001–2011 Average Annual				2008–2011 Average Annual			
California Area	Cases	Case Rank*	Rates	Rate Rank*	Cases	Case Rank*	Rates	Rate Rank*
Kern County	1327.5	1	168.5	1	1505.3	1	177.0	2
Fresno County	372.6	2	40.1	3	566.0	2	177.0	3
Los Angeles County**	171.8	3	1.8	16	237.0	4	2.4	16
Kings County	167.8	4	108.8	2	282.5	3	177.6	1
Tulare County	156.7	5	36.5	4	192.3	5	42.3	5
San Luis Obispo County**	96.3	6	36.5	5	141.8	7	44.1	4
San Diego County**	91.5	7	3.0	14	118.5	6	4.5	14
Antelope Valley**	55.8	8	14.8	7	79.0	8	18.7	7
Riverside County**	51.4	9	2.6	15	69.8	9	3.2	15
Ventura County**	43.4	10	5.3	11	48.5	12	5.8	12
Orange County**	41.7	11	1.4	18	59.0	10	1.9	17
San Joaquin County	36.7	12	5.4	10	54.3	11	7.7	10
San Bernardino County**	30.5	13	1.4	17	42.5	13	1.8	18
Monterey County**	25.9	14	6.1	9	37.0	14	8.7	9
Madera County	25.1	15	17.0	6	32.3	17	20.7	6
Stanislaus County	22.6	16	4.3	12	36.3	15	6.8	11
Merced County	20.5	17	8.1	8	34.5	16	13.0	8
Santa Clara County**	19.7	18	1.1	19	23.5	18	1.3	19
Santa Barbara County**	17.8	19	4.2	13	21.8	19	5.0	13

*Ranks are based on case counts and incidence rates of California counties. Antelope Valley, not a county but a large part of Los Angeles County, is included to demonstrate how high incidence rate can be within LA County. Compared to the 57 other counties in California during 2001–2011, LA County had the third highest average annual number of cases and Antelope Valley had a higher incidence rate than all but six counties.

**Counties not part of the Central California Valley are Los Angeles, San Luis Obispo, San Diego, Riverside, Ventura, Orange, San Bernardino, Monterey, Santa Clara, and Santa Barbara.

## Discussion

Over 1973–2011, the epidemiology of coccidioidomycosis in Los Angeles County changed. The most notable changes started in 2004. These included significant increases in case numbers and incidence rates across various demographic categories and geographic areas, a sudden and substantial rise in female cases, collectively more annual cases in the endemic areas, especially high case numbers and incidence rates in the Antelope Valley, and increasing number of case deaths in areas not known to be endemic.

The housing boom during the early and mid-2000s seems to be at least part of the initial cause for the epidemiologic changes starting in 2004. Because of the distance from large business centers, residential property in Antelope Valley is much cheaper than most parts of the county. The development of thousands of new homes in Antelope Valley was very strongly correlated to disease incidence throughout the county. With the strong high desert winds, increased construction activity in formerly undisturbed regions of the endemic area would have likely released more *Coccidioides* spores to infect previously unexposed people. Such people easily include construction workers, agricultural workers, real estate marketers, both long-term and new Antelope Valley residents, inmates of the three local correctional facilities, and visitors seeking new homes, work, and specific recreation ranging from kid soccer tournaments to dirt biking. The contrast between the gradual coccidioidomycosis increase among males starting in 2001 and the sudden sharp rise among females in 2004 suggests how the exposed population may have expanded from the male-dominated construction industry to young families seeking or moving into the new homes. Year 2005 was the peak year for newly constructed residential buildings and for male and female coccidioidomycosis cases in age groups 25–34 years and 35–44 years.

Other factors, may have contributed to the increase in coccidioidomycosis both during and after the housing boom. These include fugitive dust, drought, and expansion of agriculture in Antelope Valley. Increased usage of agricultural crops that required tilling of new fields instead of recycling fields, pulverization of soil so carrots could grow straight, and fugitive dust due to lack of proper dust control knowledge among new land owners and inability to enforce dust control ordinances on absentee land owners were reported. While adequate data to show relationships between such environmental factors and coccidioidomycosis incidence were unavailable, examination of dozens of satellite images between 2000 and 2011 found that specific sites of land development and agricultural expansion preceded nearby incident cases and clusters by at least a month. These findings are not shown because the satellite image quality is poor and further analysis with soil type data might provide more telling results. The observed agricultural expansion might be related to the much greater increase in cases among whites and Hispanics after 2004 as greater percentages of these populations work in agriculture compared to black and Asian populations[34]. The modification in CSTE coccidioidomycosis case definition to no longer require a convalescent test could have also caused cases to increase after 2008.

In general, reports of coccidioidomycosis increased substantially across the United States between 1998 and 2011 with reports coming from 28 states and the District of Columbia[35]. Recently, researchers detected *Coccidioides* in Washington State soil for the first time[36]. Other than drought and the expansion of land development and human populations into endemic areas, ecologic changes in soil and climate change might have contributed to increases in LA County and in the United States[37,38]. Between 2003 and 2005 several California counties also had an increase in cases[39]. However, none had as much of an increase as LA County during those three years. Several not-known-to-be endemic areas in LA County had much higher percent increases in coccidioidomycosis than endemic areas. Additionally, 38% of survey-responsive cases residing in areas not known to be endemic reported travelling to endemic areas outside of LA County. The collection of these findings indicates the need to educate and raise awareness of coccidioidomycosis beyond residents of known endemic areas.

Coccidioidomycosis mortality is not well-established in the literature. In 1991, Kern County conducted a clinical study of 536 coccidioidomycosis patients, 29% of whom were hospitalized and 17% had unknown outcome, and found a 3.2% one-year mortality after onset[40]. This calculation included cases who might have died from causes unrelated to the coccidioidomycosis infection, and so coccidioidomycosis mortality may be less than 3.2%. Two studies looking at non-federal hospitalizations in California estimated coccidioidomycosis mortality at 9% for 1999–2002[41] and 8% for 2000–2011[7]. These two studies analyzed hospitalized populations so less severe coccidioidomycosis cases were likely not included. With 70% of cases being reported as hospitalized and an 11% mortality in LA County, questions of missed diagnosis and non-reporting arise, particularly regarding disease not severe enough for hospital admission. Higher coccidioidomycosis mortality and the sharp increase in number of deaths during 2008–2011 in areas not known to be endemic also point to the need for better awareness among clinicians and the general public towards improved recognition, diagnosis, reporting, case management, and prevention.

Given the large number of reported cases, collaborative partnerships among Federal, State, and local government agencies and local community organizations are recommended to develop effective education and prevention strategies to protect residents and travelers. Education and awareness on a community level are vital to appropriately recognize, diagnose, treat, measure, and prevent coccidioidomycosis. In LA County, infectious disease clinicians seemingly have the highest level of coccidioidomycosis awareness among medical providers; however, general practitioners and emergency department clinicians, typically the first to see new cases, are largely unaware of the disease. In light of this, some clinicians have suggested requiring coccidioidomycosis education for new and continuing medical licensure in endemic states. Additionally, the general population needs effective education on the disease. For higher probabilities of success, education efforts and awareness campaigns should encompass input from cases and clinicians, and participation and leadership from city and neighborhood councils, local businesses and industries, schools, and other community organizations.

While population-based, this study was limited by available resources for epidemiologic investigation and passive surveillance. As described, analysis of race-ethnicity and mortality excluded years with large percentages of missing data. Inter-observer variability among the clinicians reporting disease and public health nurses conducting case investigations may have caused inconsistent sensitivity in detecting epidemiologic factors. Similarly, under-reporting and misdiagnosis, which are recognized problems even in the most endemic areas are unmeasured and likely present. Because LA County has had mandatory laboratory reporting for coccidioidomycosis, the 2009 requirement for laboratory reporting to the state of California is not considered a major contributing factor for the observed trend of increasing disease, especially with the trend starting in 2004. In 2009, all case interviews ended and the main source for exposure data became the medical chart. As such, exposure data before 2009 likely represents people who were reachable by telephone during work hours of week days. The true burden of disease is underestimated also because surveillance excludes previously reported cases, a few of which may be re-infections or re-activations several months or years after prior infection.

The authors present this study to help raise awareness and inform government and community planning efforts to prevent unnecessary coccidioidomycosis disease and mortality. Effective education and awareness efforts beyond medical communities of Central California and Arizona are needed. Engaging local community organizations and local government agencies to collaborate in endemic areas and areas of low or unrecognized endemicity but with a history of cases is a progressive step towards better public health. LA County serves as an example of how coccidioidomycosis can potentially increase. Land development in previously undisturbed endemic areas and increased population exposure can lead to the ongoing experience of substantially more coccidioidomycosis cases and changed epidemiologic profile. In 2012 and 2013, LA County had 327 and 362 confirmed cases, respectively, but another steep increase in cases such as in 2004 could occur if development in endemic areas extensively exposes resident, working, or visiting populations. Development can involve any soil-disturbing activity including environmental cleanup. Other than epidemiologic data, ecologic studies with soil testing and geological maps on soil type can inform development planning when soil disturbance is proposed in endemic areas and nearby areas not known to be endemic for *Coccidioides*[37]. Planning should involve local residential communities and businesses when developing and executing education and prevention strategies to minimize exposure of residents and traveling visitors.

## Supporting Information

S1 Fig Coccidioidomycosis cases among 0–14 and 15–24 year-olds by gender, Los Angeles County, California, 1992–2011. Legend. Differences in 2004–2011 included more cases, annual cases among females at 0–14 years-old, and a general increasing trend for male 15–24 year-olds.
(TIF)

S2 Fig Coccidioidomycosis cases among 25–34 and 35–44 year-olds by gender, Los Angeles County, California, 1992–2011. Legend. Unlike in other age groups, peak incidence occurred in 2005.
(TIF)

S3 Fig Coccidioidomycosis cases among 45–54 and 55–64 year-olds by gender, Los Angeles County, California, 1992–2011. Legend. All four demographic groups displayed a trend of increasing annual cases after 2003.
(TIF)

S4 Fig Coccidioidomycosis cases among ≥65 year-olds by gender, Los Angeles County, California, 1992–2011. Legend. A general trend of increasing annual cases occurred for both demographic groups during 2004–2011 with males experiencing a pronounced spike in 2011.
(TIF)

S1 Table Number of coccidioidomycosis cases (N = 2530) by age and gender, Los Angeles County, 1992–2011. Legend. Thirteen cases missing age or gender are not included. In all age groups, females had fewer cases than males. Within age groups, incidence trends of females generally reflected those of males. During 1992–2011, the greatest number of male cases occurred in the 35–44 and 45–54 year age groups and the greatest number of female cases occurred in the 45–54 year age group.
(DOCX)
