# Supplementary figures and images for: The Changing Epidemiology of Coccidioidomycosis in Los Angeles (LA) County, California, 1973–2011
Source: PLoS One. 2015 Aug 27;10(8):e0136753. doi: 10.1371/journal.pone.0136753 (PMC4551673; doi:10.1371/journal.pone.0136753)

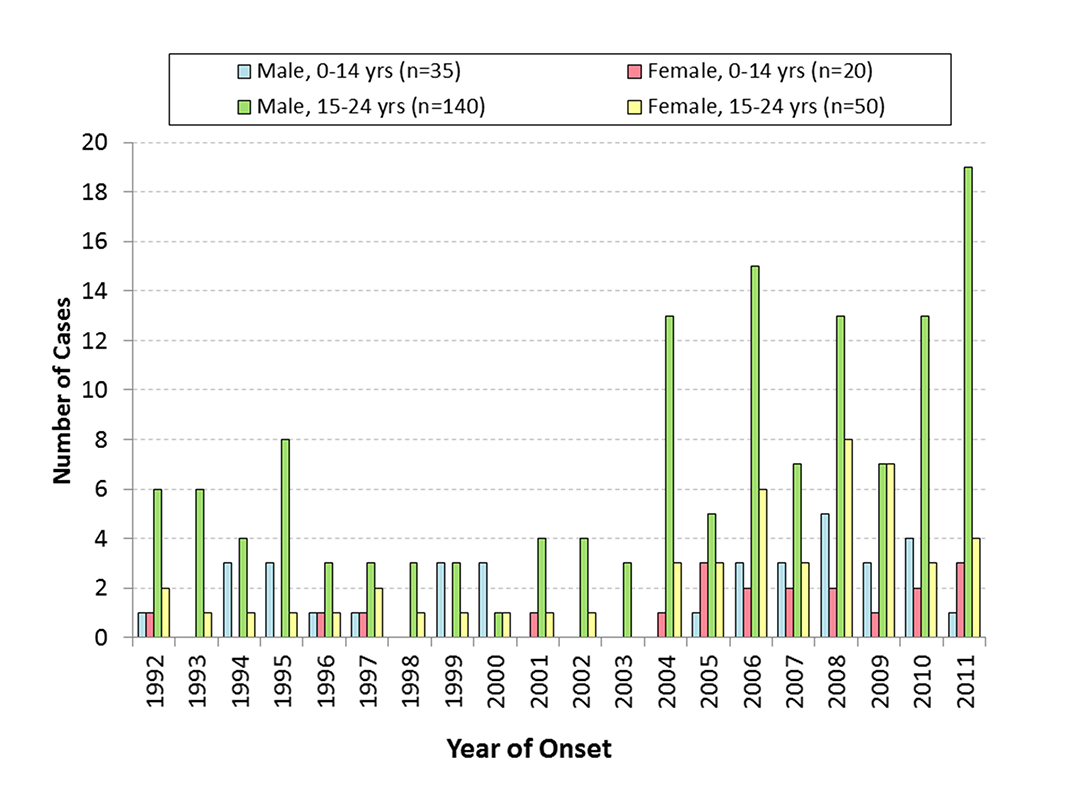

Supplement: S1 Fig — Legend. Differences in 2004–2011 included more cases, annual cases among females at 0–14 years-old, and a general increasing trend for male 15–24 year-olds. (TIF) [file pone.0136753.s001.TIF]

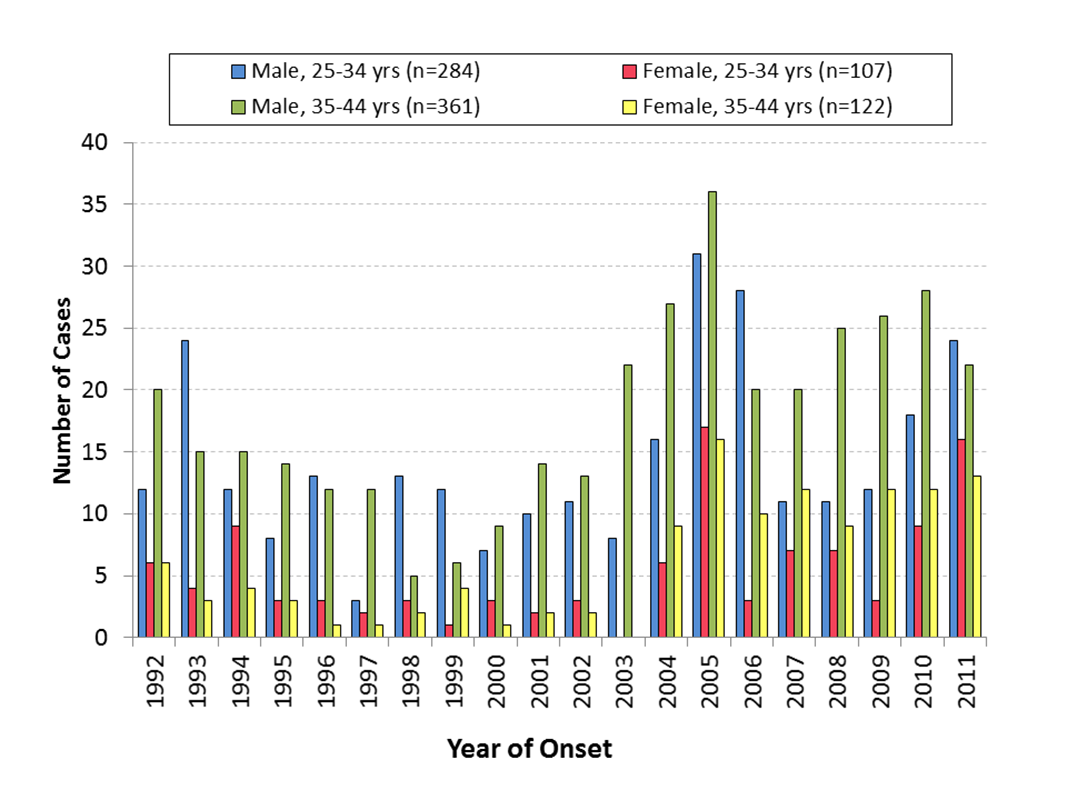

Supplement: S2 Fig — Legend. Unlike in other age groups, peak incidence occurred in 2005. (TIF) [file pone.0136753.s002.TIF]

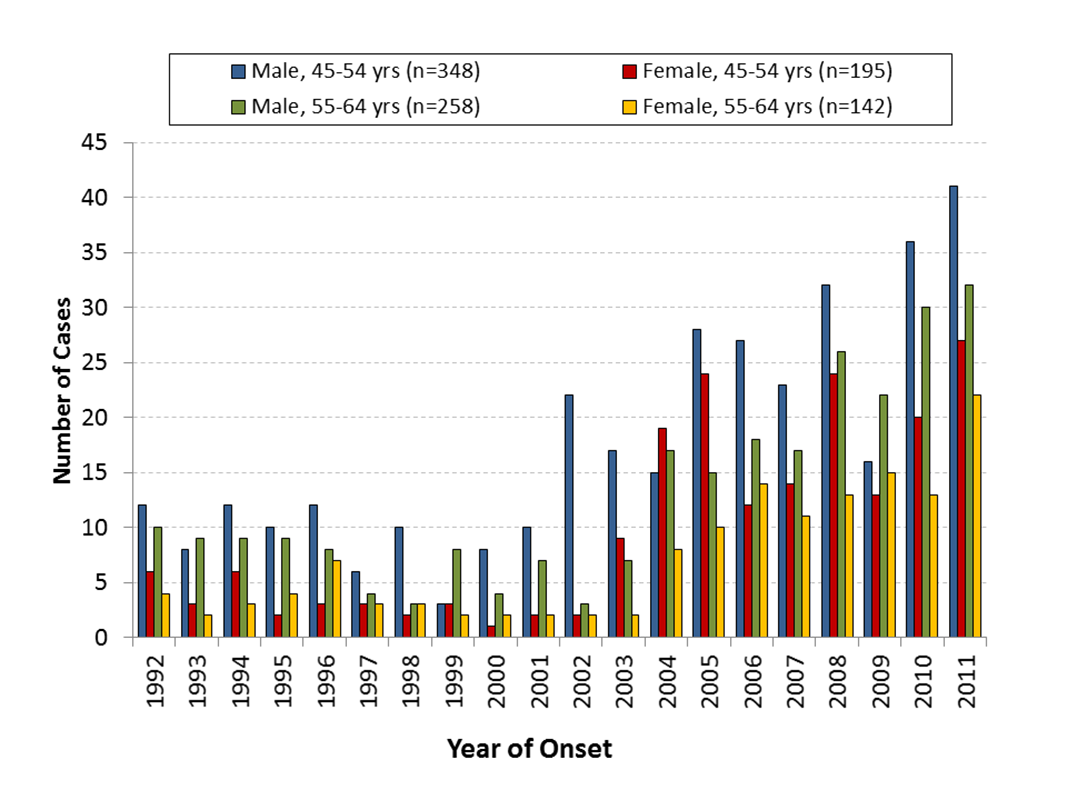

Supplement: S3 Fig — Legend. All four demographic groups displayed a trend of increasing annual cases after 2003. (TIF) [file pone.0136753.s003.TIF]

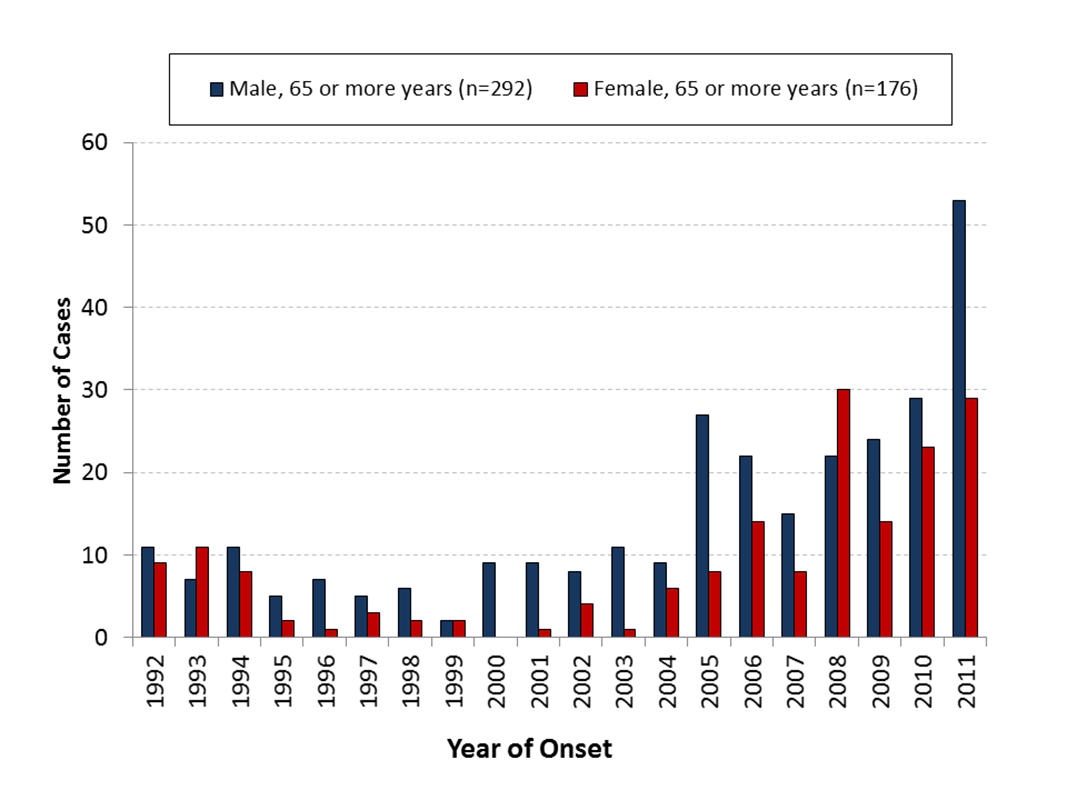

Supplement: S4 Fig — Legend. A general trend of increasing annual cases occurred for both demographic groups during 2004–2011 with males experiencing a pronounced spike in 2011. (TIF) [file pone.0136753.s004.TIF]
