# Supplementary material for: The Changing Epidemiology of Coccidioidomycosis in Los Angeles (LA) County, California, 1973–2011
Source: PLoS One. 2015 Aug 27;10(8):e0136753. doi: 10.1371/journal.pone.0136753 (PMC4551673; doi:10.1371/journal.pone.0136753)
Supplement: S1 Table — Legend. Thirteen cases missing age or gender are not included. In all age groups, females had fewer cases than males. Within age groups, incidence trends of females generally reflected those of males. During 1992–2011, the greatest number of male cases occurred in the 35–44 and 45–54 year age groups and the greatest number of female cases occurred in the 45–54 year age group. (DOCX) [file pone.0136753.s005.docx]

**S1 Table. Number of coccidioidomycosis cases (N=2530) by age and gender, Los Angeles County, 1992-2011.**

| **Year of Onset** | **Age Group (years)** | | | | | | | | | | | | | |  |
| --- | --- | --- | --- | --- | --- | --- | --- | --- | --- | --- | --- | --- | --- | --- | --- |
|  | **0-14** | | **15-24** | | **25-34** | | **35-44** | | **45-54** | | **55-64** | | **≥65** | | **Total** |
|  | **Male** | **Female** | **Male** | **Female** | **Male** | **Female** | **Male** | **Female** | **Male** | **Female** | **Male** | **Female** | **Male** | **Female** |  |
| 1992 | 1 | 1 | 6 | 2 | 12 | 6 | 20 | 6 | 12 | 6 | 10 | 4 | 11 | 9 | 106 |
| 1993 | 0 | 0 | 6 | 1 | 24 | 4 | 15 | 3 | 8 | 3 | 9 | 2 | 7 | 11 | 93 |
| 1994 | 3 | 0 | 4 | 1 | 12 | 9 | 15 | 4 | 12 | 6 | 9 | 3 | 11 | 8 | 97 |
| 1995 | 3 | 0 | 8 | 1 | 8 | 3 | 14 | 3 | 10 | 2 | 9 | 4 | 5 | 2 | 72 |
| 1996 | 1 | 1 | 3 | 1 | 13 | 3 | 12 | 1 | 12 | 3 | 8 | 7 | 7 | 1 | 73 |
| 1997 | 1 | 1 | 3 | 2 | 3 | 2 | 12 | 1 | 6 | 3 | 4 | 3 | 5 | 3 | 49 |
| 1998 | 0 | 0 | 3 | 1 | 13 | 3 | 5 | 2 | 10 | 2 | 3 | 3 | 6 | 2 | 53 |
| 1999 | 3 | 0 | 3 | 1 | 12 | 1 | 6 | 4 | 3 | 3 | 8 | 2 | 2 | 2 | 50 |
| 2000 | 3 | 0 | 1 | 1 | 7 | 3 | 9 | 1 | 8 | 1 | 4 | 2 | 9 | 0 | 49 |
| 2001 | 0 | 1 | 4 | 1 | 10 | 2 | 14 | 2 | 10 | 2 | 7 | 2 | 9 | 1 | 65 |
| 2002 | 0 | 0 | 4 | 1 | 11 | 3 | 13 | 2 | 22 | 2 | 3 | 2 | 8 | 4 | 75 |
| 2003 | 0 | 0 | 3 | 0 | 8 | 0 | 22 | 0 | 17 | 9 | 7 | 2 | 11 | 1 | 80 |
| 2004 | 0 | 1 | 13 | 3 | 16 | 6 | 27 | 9 | 15 | 19 | 17 | 8 | 9 | 6 | 149 |
| 2005 | 1 | 3 | 5 | 3 | 31 | 17 | 36 | 16 | 28 | 24 | 15 | 10 | 27 | 8 | 224 |
| 2006 | 3 | 2 | 15 | 6 | 28 | 3 | 20 | 10 | 27 | 12 | 18 | 14 | 22 | 14 | 194 |
| 2007 | 3 | 2 | 7 | 3 | 11 | 7 | 20 | 12 | 23 | 14 | 17 | 11 | 15 | 8 | 153 |
| 2008 | 5 | 2 | 13 | 8 | 11 | 7 | 25 | 9 | 32 | 24 | 26 | 13 | 22 | 30 | 227 |
| 2009 | 3 | 1 | 7 | 7 | 12 | 3 | 26 | 12 | 16 | 13 | 22 | 15 | 24 | 14 | 175 |
| 2010 | 4 | 2 | 13 | 3 | 18 | 9 | 28 | 12 | 36 | 20 | 30 | 13 | 29 | 23 | 240 |
| 2011 | 1 | 3 | 19 | 4 | 24 | 16 | 22 | 13 | 41 | 27 | 32 | 22 | 53 | 29 | 306 |
| Total | 35 | 20 | 140 | 50 | 284 | 107 | 361 | 122 | 348 | 195 | 258 | 142 | 292 | 176 | 2530 |

Thirteen cases missing age or gender are not included. In all age groups, females had fewer cases than males. Within age groups, incidence trends of females generally reflected those of males. During 1992-2011, the greatest number of male cases occurred in the 35-44 and 45-54 year age groups and the greatest number of female cases occurred in the 45-54 year age group.
